# α-Mangostin: A Xanthone Derivative in Mangosteen with Potent Anti-Cancer Properties

**DOI:** 10.3390/biom14111382

**Published:** 2024-10-30

**Authors:** Amin F. Majdalawieh, Tala M. Terro, Sogand H. Ahari, Imad A. Abu-Yousef

**Affiliations:** Department of Biology, Chemistry, and Environmental Sciences, College of Arts and Sciences, American University of Sharjah, Sharjah P.O. Box 26666, United Arab Emirates; g00086819@alumni.aus.edu (T.M.T.); g00087587@alumni.aus.edu (S.H.A.); iabuyousef@aus.edu (I.A.A.-Y.)

**Keywords:** α-mangostin, anti-cancer, xanthone derivative, antioxidant, anti-proliferative

## Abstract

α-Mangostin, a xanthone derivative extracted from the pericarp of the mangosteen fruit (*Garcinia mangostana* L.), has garnered significant attention for its potential as a natural anti-cancer agent. This review provides a comprehensive analysis of the current literature on the anti-cancer properties of α-mangostin across various cancer types. Through an extensive analysis of in vitro and in vivo studies, this review elucidates the multifaceted mechanisms underlying α-mangostin’s cytotoxicity, apoptosis induction through both intrinsic and extrinsic pathways, and modulation of key cellular processes implicated in cancer progression in a diverse array of cancer cells. It causes mitochondrial dysfunction, activates caspases, and regulates autophagy, endoplasmic reticulum stress, and oxidative stress, enhancing its anti-cancer efficacy. Moreover, α-mangostin exhibits synergistic effects with conventional chemotherapeutic agents, suggesting its utility in combination therapies. The ability of α-mangostin to inhibit cell proliferation, modulate cell cycle progression, and induce apoptosis is linked to its effects on key signaling pathways, including Akt, NF-κB, and p53. Preclinical studies highlight the therapeutic potential and safety profile of α-mangostin, demonstrating significant tumor growth inhibition without adverse effects on normal cells. In summary, understanding the molecular targets and mechanisms of action of α-mangostin is crucial for its development as a novel chemotherapeutic agent, and future clinical investigations are warranted to explore its clinical utility and efficacy in cancer prevention and therapy.

## 1. Introduction

Cancer remains one of the most formidable challenges in modern medicine, with its complex etiology and heterogeneous nature requiring innovative therapeutic approaches. Studies indicate that the global cancer burden is expected to rise significantly, with new cancer cases projected to increase from 18.1 million in 2018 to 29.5 million by 2040 [1]. Concurrently, cancer-related deaths are anticipated to grow from 9.6 million to 16.4 million annually [1]. Despite advancements in conventional therapies, such as surgery, radiation, and chemotherapy, these treatments often come with significant toxicity, high costs, and limited long-term efficacy. This has driven the exploration and integration of natural compounds as potential anti-cancer agents due to their diverse bioactive properties and generally lower toxicity profiles compared with conventional treatments like chemotherapy [2].

Research published in leading journals such as Cancer Research and Oncogene has demonstrated the efficacy of various plant-derived compounds in targeting specific pathways involved in tumor growth and progression, such as counteracting free radicals, reducing oxidative stress, inhibiting cancer cell proliferation, inducing apoptosis, and suppressing metastasis [3,4]. Furthermore, the development of natural compounds as adjuncts to standard cancer treatments has shown promising results [5]. By combining these compounds with conventional therapies, such as chemotherapy or radiation, researchers aim to enhance treatment outcomes while reducing toxicity to healthy cells [5].

Among these natural compounds, α-mangostin, derived from the pericarp of the mangosteen fruit (*Garcinia mangostana* L.) as well as other plants (e.g., *Hydnocarpus octandra* and *Hydnocarpus venenatus*), has emerged as a promising bioactive candidate with significant anti-cancer potential. Its chemical formula is C_24_H_26_O_6_, and it has a molecular weight of 410.46 g/mol. The compound is known for its poor solubility in water but is soluble in organic solvents such as ethanol, methanol, and dimethyl sulfoxide (DMSO). With a melting point of around 180–181 °C, the chemical properties of α-mangostin play a crucial role in its bioactivity, especially in the context of therapeutic applications. α-Mangostin, known for its wide range of therapeutic benefits, has been traditionally used in Southeast Asian medicine for diarrhea, chronic ulcers, skin infection treatments, and wounds for centuries [6]. Some of the anti-cancer properties of α-mangostin are attributed to its anti-proliferative [7], pro-apoptotic [8], anti-metastatic [9], anti-angiogenic [10], and antioxidant [11] activities, while regulating a number of signal transduction pathways that mediate these processes. α-Mangostin has a wide range of biological actions, including, but not limited to, increasing the activity of caspase-3, caspase-8, and caspase-9, modulating cell cycle regulators, increasing the B-cell lymphoma 2 (Bcl-2) and Bcl-2-associated X protein (Bax/Bcl-2) ratio, increasing reactive oxygen species (ROS) levels, inhibiting the phosphoinositide 3-kinase (PI3K) / protein kinase B (Akt) and nuclear factor kappa-light-chain-enhancer of activated B cells (NF-κB) signaling pathways, and reducing tumor volume in animal models [8,11,12,13,14]. These anti-carcinogenic effects of α-mangostin enable it to suppress the effects of cancer-inducing compounds. This is mainly achieved by directly targeting transformed cancerous cells or by blocking cellular mechanisms that are crucial for promoting tumorigenesis.

This review aims to comprehensively summarize the existing body of literature on the anti-cancer properties of α-mangostin across various cancer types, including, but not limited to, colon cancer, glioblastoma, melanoma, oral squamous cell carcinoma, and pancreatic cancer. By compiling data from in vitro and in vivo studies, we seek to elucidate the molecular mechanisms underlying the anti-cancer effects of α-mangostin and its potential as a novel therapeutic agent. The review encompasses studies investigating the effects of α-mangostin on cell proliferation, apoptosis induction, cell cycle regulation, metastasis inhibition, and modulation of key signaling pathways implicated in cancer progression.

## 2. Anti-Proliferative and Pro-Apoptotic Effects of α-Mangostin

α-Mangostin exhibits strong anti-proliferative and pro-apoptotic effects on various cancer cells. Through in vitro and in vivo studies, we detail how α-mangostin impacts key cellular processes to suppress tumor growth and promote cell death. Several studies have explored the anti-cancer potential of α-mangostin in various breast cancer cells, shedding light on its mechanisms of action and therapeutic implications. Kurose and colleagues conducted an in vitro investigation of the effects of α-mangostin on apoptosis and cell cycle regulation in MDA-MB231 cells, finding that α-mangostin induced mitochondria-mediated apoptosis and growth phase-1 (G1) arrest at a concentration of 20 μM [7]. Additionally, α-mangostin treatment upregulated p21cip1 expression while downregulating cell cycle progression proteins, indicating its ability to modulate cell cycle regulators. Further confirmation of apoptosis induction was observed through elevated levels of single-stranded deoxyribonucleic acid (DNA) and increased activity of caspase-3, caspase-8, and caspase-9 in MDA-MB231 cells treated with α-mangostin (20 μM) for 24 h. Moreover, levels of cytochrome c protein in mitochondrial fractions were significantly reduced in α-mangostin-treated cells, indicating its release from mitochondria and thereby implicating the involvement of the mitochondria-mediated apoptotic pathway. Concurrently, Kritsanawong and colleagues demonstrated that α-mangostin (7.5, 15, and 30 μM) causes a dose- and time-dependent inhibition of colony formation and induction of apoptosis in T47D cells [12]. α-Mangostin (7.5, 15, and 30 μM) reduced myeloid cell leukemia sequence-1 (Mcl-1) expression without affecting B-cell lymphoma-extra large (Bcl-xL) and Bax expression, resulting in a significant increase in the Bax/Bcl-2 ratio. Additionally, treatment with 30 μM α-mangostin also led to an increase in Bax oligomers, causing mitochondrial membrane permeability changes and subsequent release of cytochrome c into the cytosol in T47D cells. JC-1 staining revealed a higher green/red fluorescence ratio indicative of mitochondrial depolarization, confirming the loss of mitochondrial membrane potential induced by α-mangostin treatment. Ibrahim and colleagues explored α-mangostin-induced apoptosis in MCF-7 and LA7 cells, highlighting concentration-dependent apoptotic features, mitochondrial dysfunction, and caspase-3, caspase-7, caspase-8, and caspase-9 activation [13]. α-Mangostin (5, 10, and 20 μg/mL) treatment upregulated pro-apoptotic protein Bax while downregulating anti-apoptotic protein Bcl-2, resulting in an increased Bax/Bcl-2 ratio, favoring apoptosis. Additionally, α-mangostin (5, 10, and 20 μg/mL) treatment inhibited cell proliferation and induced poly ADP-ribose polymerase (PARP) cleavage, suggesting inhibition of cell proliferation and induction of apoptosis. The in vivo experiments in rats showed that α-mangostin inhibited tumor growth at doses of 30 and 60 mg/kg without affecting body weight. The reduction in tumor volume was greater with the higher dose of α-mangostin (60 mg/kg) compared with the lower dose (30 mg/kg) [13]. Simon and colleagues further elucidated the apoptotic effects of α-mangostin in MCF-7 cells, mediated by upregulating endogenous modulator of apoptosis-1 (MOAP-1) and downregulating Bcl-xL proteins [8]. Another study investigated the effects of α-mangostin (5, 10, and 20 μg/mL) on breast cancer spheroids, showing dose-dependent effects on morphology and viability, as well as its ability to reduce retinaldehyde-dependent aldehyde dehydrogenase (RALDH) activity [15]. Won and colleagues revealed α-mangostin’s (1, 5, and 10 μM) inhibition of cell growth and induction of apoptosis in MCF-7 estrogen receptor a (ERα)-positive cells, implicating caspase-dependent and -independent pathways and downregulation of Erα [16]. Moreover, Shibata and colleagues investigated the anti-tumor and anti-metastatic effects of α-mangostin in mammary cancer cell lines (BJMC3879luc2 and MDA-MB231) and female BALB/c mice [17]. α-Mangostin treatment (12 μM) induced apoptosis, evidenced by elevated caspase-3, caspase-8, and caspase-9 activity and cytochrome c release, while also causing G1-phase cell cycle arrest. In vivo experiments demonstrated significant tumor growth inhibition and increased survival rates in α-mangostin-treated mice. Interestingly, immunohistochemistry also showed a markedly lower level of p-Akt (Thr^308^) in mammary carcinomas of mice treated with α-mangostin (20 mg/kg/day) compared with control tumors. Moreover, Han and colleagues identified α-mangostin (0.62, 1.25, and 2.50 μM) as a potent inhibitor of lysine-specific demethylase 1 (LSD1), a histone demethylase enzyme, and as a scaffold for assembling chromatin modifier and transcription factor complexes to regulate gene expression in breast cancer cells, suggesting a novel avenue for LSD1 inhibitor research [18]. They also reported that α-mangostin increased H3K4me2, a histone post-translational modification enriched in *cis*-regulatory regions of transcriptionally active genes as well as genes primed for future expression during cell development, accumulation and CD86 expression [18]. Scolamiero and colleagues investigated the effects of α-mangostin (0.1, 0.5, 1, 5, 10, 15, 20, and 30 μg/mL) on multicellular tumor spheroids (MCTSs) derived from MDA-MB-231 and MCF-7 breast tumor cells, observing dose-dependent decreases in cell viability and induction of apoptosis [19]. Additionally, α-mangostin affected the size and consistency of spheroids, with higher concentrations leading to reduced volume and increased compactness. Li and colleagues demonstrated α-mangostin’s dose- and time-dependent cytotoxic effects on breast cancer cells, inducing apoptosis characterized by increased PARP cleavage and alterations in the Bax/Bcl-2 ratio [20]. Huang and colleagues explored α-mangostin’s (1, 2, and 4 μM) induction of autophagy and endoplasmic reticulum (ER) stress in breast cancer cells, as evidenced by the formation of autophagic vesicles and increased expression of microtubule-associated protein-1 light chain 3 (LC3II/LC3I) and p62 proteins, markers of autophagy [21].

Several studies have investigated the cytotoxic effects of α-mangostin on A549 cells, revealing its potential as a therapeutic agent against non-small cell lung cancer (NSCLC). Ding and colleagues explored α-mangostin’s cytotoxicity, demonstrating its concentration-dependent inhibition of A549 cell proliferation, induction of apoptosis, and alteration of cell cycle distribution [22]. α-Mangostin (above 6 μg/mL) treatment promoted ROS production through downregulation of nicotinamide phosphoribosyltransferase (NAMPT) / nicotinamide adenine dinucleotide (NAD) and significant damage to mitochondrial membrane integrity, implicating its role in inducing apoptosis and inhibiting cell proliferation in A549 cells. Similarly, Phan and colleagues observed α-mangostin’s (1–100 µM) dose- and time-dependent inhibition of A549 cell viability and growth, with cytotoxic effects observed at higher concentrations [23]. In contrast, lower concentrations of α-mangostin were non-toxic to both lung cancer cells and normal cells. Additionally, Zhang and colleagues investigated α-mangostin’s (5 and 10 μM) therapeutic potential against A549 cells, noting its dose-dependent decrease in cell viability, induction of apoptosis, inhibition of cell migration, and increase in ROS generation as evidenced by annexin V/propidium iodide (PI) staining and increased Bax/Bcl-2 ratio [24]. Furthermore, Ding and colleagues elucidated α-mangostin’s (2, 4, 6, 8, 10, and 12 μg/mL) cytotoxicity mechanisms in A549 cells, highlighting its ability to induce apoptosis, oxidative stress, and cell cycle arrest in a concentration-dependent manner, with p38 and p53 phosphorylation playing a role in mediating cell cycle arrest, affecting cyclin D1 and cyclin-dependent kinase 4 (CDK4) expression [22]. α-Mangostin (2, 4, 6, 8, 10, and 12 μg/mL) treatment also reduced intracellular NAD levels and suppressed NAMPT expression, contributing to ROS accumulation and activation of the mitochondrial apoptosis pathway in A549 cells [22]. These findings collectively underscore α-mangostin’s potential as a therapeutic agent for NSCLC treatment, warranting further investigation into its clinical utility.

Several studies have investigated the apoptotic effects of α-mangostin on osteosarcoma cells, shedding light on its potential therapeutic value in treating this malignancy. Yang and colleagues conducted comprehensive in vitro and in vivo studies to elucidate α-mangostin’s (10, 20, 30, 40, and 50 μM) apoptotic mechanisms in human osteosarcoma cells through ROS-mediated ER stress via the Wnt pathway [25]. α-Mangostin (10, 20, 30, 40, and 50 μM) treatment exhibited concentration- and time-dependent suppression of viability in 143B and Saos-2 osteosarcoma cells, inducing apoptosis and increasing ROS production without affecting normal osteoblasts. Activation of the caspase-3/caspase-8 cascade and upregulation of CCAAt-enhancer binding proteins (C/EBP) homologous protein (CHOP) and activating transcription factor 6 (ATF6) expression were observed, indicating the involvement of ROS-mediated ER stress via the Wnt pathway. In vivo studies using female athymic BALB/c nude mice supported these findings, with intraperitoneal α-mangostin administration leading to significant suppression of tumor volume and weight, accompanied by upregulation of cleaved caspase-3 and caspase-8 expression in tumor tissues. Similarly, Park and colleagues investigated α-mangostin’s (2.5, 5, 10, 15, 20, 30, 40, and 50 μM) therapeutic potential in MG-63 cells, demonstrating a dose-dependent decrease in cell viability and induction of apoptosis characterized by nuclear condensation and increased sub-G1 fraction [26]. α-Mangostin (10 and 25 μM) disrupted mitochondrial membrane potential as evidenced by altered expression of Bcl-2 and Bcl-2 homologous antagonist killer (Bak) proteins, collapse of mitochondrial membrane potential, and release of cytochrome c. Additionally, Krajarng and colleagues explored α-mangostin’s (5, 10, 15, 20, and 30 μg/mL) effects on canine osteosarcoma D-17 cells, revealing a dose-dependent decrease in cell viability and induction of apoptosis characterized by nuclear condensation, DNA fragmentation, and altered mitochondrial membrane potential [27]. These studies collectively highlight α-mangostin’s potential as a promising therapeutic agent for osteosarcoma treatment, warranting further investigation into its clinical application.

Lee and colleagues conducted comprehensive investigations into the anti-cancer properties of α-mangostin in cervical cancer cells, encompassing both in vitro and in vivo studies [28]. α-Mangostin (10, 20, and 30 μM) demonstrated a dose-dependent reduction in cell viability and induced apoptosis in HeLa and SiHa cells, activating both apoptotic and necrotic cell death pathways. Treatment with α-mangostin (10, 20, and 30 μM) led to the activation of apoptotic markers such as the cleaved forms of caspase-3, caspase-9, and PARP. Furthermore, α-mangostin (10, 20, and 30 μM) disrupted mitochondrial membrane potential, increased Bax and cytochrome c expression, and decreased Bcl-2 expression, indicative of activation of the mitochondrial apoptotic pathway. Additionally, α-mangostin (10, 20, and 30 μM) induced apoptotic cell death by activating the p38 signaling pathway, as evidenced by increased phosphorylation of p38 and enhanced ROS production. In vivo studies corroborated these findings, with α-mangostin administration (20 and 40 mg/kg) inhibiting tumor growth in a mouse model of cervical cancer by activating phosphorylated apoptosis signal-regulating kinase-1 (p-ASK1), p-p38, cleaved caspase-3, and cleaved PARP in tumor tissues without affecting body weight. Similarly, Díaz and colleagues investigated the effects of α-mangostin on cervical cancer cell lines (C33a, HeLa, SiHa, and CaSki) and tumor growth inhibition both in vitro and in vivo [29]. α-Mangostin (1–10 µM) inhibited cell proliferation in a concentration-dependent manner across all tested cell lines, with the most significant effect observed in CaSki cells, which harbored the highest number of human papillomavirus (HPV) copies. α-Mangostin (10 µM) induced cell cycle arrest and decreased the expression of HPV16 oncogenes (E6 and E7) and potassium voltage-gated channel subfamily H member 1 (KCNH1), whose overexpression was found to be associated with osteosarcoma pathogenesis in xenografted SiHa cells. α-mangostin (8 mg/kg) significantly reduced tumor growth and inhibited the expression of E6, E7, and KCNH1 while increasing Kiel 67 (Ki-67) expression compared with vehicle-treated female athymic nude mice (BALB/c). The effects of α-mangostin on cancer stem cells (CSCs) in cervical cancer cell lines (SiHa and HeLa) were also investigated by Chien and colleagues [30]. CSCs are known for their tumor-initiating abilities and resistance to chemotherapy. α-Mangostin (10, 20, and 30 μM) reduced cell viability, sphere-forming ability, and stemness marker expression, inducing apoptosis and mitochondrial depolarization. In vivo, orally administered α-mangostin in male BALB/c nude mice also reduced tumor growth.

In an in vitro study by Shan and colleagues, the anti-tumor effects of α-mangostin on human gastric adenocarcinoma cell lines (BGC-823 and SGC-7901) were investigated [31]. α-Mangostin (1–10 μg/mL) demonstrated a dose- and time-dependent inhibition of cell proliferation in gastric adenocarcinoma cells, with significant reductions in cell viability observed at higher concentrations. Additionally, α-mangostin (7 μg/mL) induced apoptosis in both cell lines, as evidenced by increased apoptotic rates observed through flow cytometry. Transmission electron microscopy (TEM) further confirmed apoptotic features such as chromatin condensation and apoptotic body formation. Li and colleagues explored the impact of α-mangostin on chemoresistance in gastric cancer cell lines (SGC7901 and SGC7901/CDDP) [32]. Treatment with α-mangostin (10, 15, 20, 25, and 30 μM) resulted in reduced cell viability, sensitizing SGC7901/CDDP cells to cisplatin (CDDP) and enhancing apoptosis, indicating its potential as a chemosensitizer in gastric cancer therapy. Furthermore, α-mangostin (10, 15, 20, 25, and 30 μM) treatment increased levels of autophagy-related proteins, LC3-II/I and Beclin1, while decreasing p62 levels, suggesting enhanced autophagy induction.

Several studies have investigated the anti-cancer effects of α-mangostin on colorectal cancer. Watanapokasin and colleagues conducted an in vitro study on human colon cancer cell lines (COLO 205, MIP-101, and SW620), revealing that α-mangostin (30 μg/mL) treatment induced concentration- and time-dependent decreases in cell viability, accompanied by apoptotic morphological changes including cell rounding, blebbing, and apoptotic body formation indicative of apoptosis observed after 3, 6, 9, and 12 h of treatment and activation of caspase-3, caspase-8, and caspase-9 [33]. The induction of apoptosis by α-mangostin (10, 20, and 30 μg/mL) was also accompanied by increased p-p53, pro-apoptotic Bax and Bcl-2-modifying factor (Bmf), and the release of cytochrome c from the mitochondria to the cytosol. Upregulation of BH3 interacting-domain death agonist (Bid), truncated-Bid (t-Bid), and Fas receptor (FasR) further supported the apoptotic effects of α-mangostin (10, 20, and 30 μg/mL). Kumazaki and colleagues explored α-mangostin’s impact on human colon cancer cell lines (DLD-1, SW480, and COLO201), demonstrating a concentration-dependent suppression of cell growth mediated primarily through apoptosis, with involvement of caspase-9 activation and PARP-1 cleavage [34]. α-Mangostin (5, 10, and 20 μM) also suppressed the PI3K/Akt signaling pathway and inactivated mitogen-activated protein kinase (MAPK) pathways, including extracellular signal-regulated kinase (ERK1/2), jun N-terminal kinase (JNK), and p38. Moreover, Matsumoto and colleagues studied α-mangostin’s anti-proliferative effects on human colon cancer DLD-1 cells in vitro, observing inhibition of cell JNK, growth, induction of apoptosis, and upregulation of p27 levels while downregulating cyclins and CDK1, leading to G1-phase cell cycle arrest [35]. Jo and colleagues assessed α-mangostin’s inhibitory effects on colorectal CSCs, demonstrating its ability to reduce sphere formation dose-dependently and selectively target CSCs without cytotoxicity [36]. In vivo, in male athymic BALB/c mice, intraperitoneally administered α-mangostin reduced the proportion of cluster of differentiation 133+/44+ (CD133+CD44+) CSCs in excised tumors compared with control. Aisha and colleagues investigated α-mangostin’s potential for colorectal carcinoma treatment, highlighting its significant cytotoxicity against colorectal carcinoma cell lines (HCT-116 and CCD-18Co) while exhibiting lower cytotoxicity towards normal colonic cells [37]. α-Mangostin (2.5, 5, 7.5, 10, and 12.5 µg/mL) enhanced p53/DNA damage, Myc/Myc-associated factor X (Max), and MAPK/ERK signaling pathways while downregulating the NF-κB pathway. Additionally, Kim and colleagues found that α-mangostin inhibited colon cancer growth by inducing autophagy in mouse intestinal epithelial cell lines (CT26 and Her-2/neu) [38]. In vitro, α-mangostin (100 and 250 ng/mL) reduced ER stress induced by thapsigargin and inhibited eukaryotic initiation factor 2a (eIF2α) phosphorylation. In vivo, α-mangostin (20 mg/kg) induced autophagy activation and apoptotic effect in intestinal epithelial cells with thapsigargin-like calcium^2+^ (Ca^2+^)-adenosine triphosphase(ATP)ase inhibitory effect without significant T cell proliferation. Lee and colleagues explored α-mangostin’s ability to sensitize 5-FU-resistant colon cancer cells (SNUC5 and SNUC5/5-FUR) to apoptosis [39]. α-Mangostin (20 μM) increased the formation of intracellular oxidative damages, including 8-hydroxyguanosine (8-OH-G) and 4-hydroxynonenal (4-HNE). α-Mangostin (20 μM) also induced apoptosis in the cells through activation of both extrinsic and intrinsic pathways, as evidenced by the activation of caspases and PARP, along with cytochrome c release. Importantly, the expression of FasR, critical for α-mangostin-induced apoptosis, was lower in the 5-FU-resistant cells compared with the susceptible ones. Finally, Nakagawa and colleagues demonstrated α-mangostin’s cytotoxicity against DLD-1 cells in a concentration-dependent manner [40]. α-Mangostin induced apoptotic cell death at concentrations exceeding 15 μM, characterized by perturbation of mitochondrial membrane potential and increased release of endonuclease-G from mitochondria, as well as increased microRNA-143 (miR-143) levels and reduced ERK5 expression. Interestingly, caspase activation was not involved in α-mangostin-induced apoptosis, suggesting the involvement of alternative apoptotic pathways.

Shi and colleagues evaluated the efficacy of α-mangostin in inhibiting gallbladder cancer (GBC) cell lines (GBC-SD, HIBEC, and NOZ) growth and enhancing sensitivity to chemotherapy [41]. α-Mangostin (1, 2, 4, 6, 8, 12, and 16 μM) treatment significantly reduced GBC cell growth, suppressed colony formation and proliferation, induced apoptosis, and cell cycle arrest in a dose- and time-dependent manner. In vivo experiments in mice further confirmed α-mangostin’s (2 mg/kg) potential in reducing tumor growth. Li and colleagues investigated the potential of α-mangostin to modulate ER stress proteins in prostate cancer cell lines (22Rv1 and LNCaP) and prostate epithelial cells from patients [20]. α-Mangostin (7.5 and 15 μM) reduced cell proliferation and increased cleaved caspase-3 levels in a dose-dependent manner, indicating induction of apoptosis. α-Mangostin (7.5 and 15 μM) also increased expression of ER stress markers in prostate cancer cells, suggesting the promotion of ER stress. However, in non-tumorigenic prostate epithelial cells, α-mangostin (15 μM) reduced protein kinase RNA-like ER kinase (PERK) levels and did not increase CHOP, indicating a differential effect on ER stress proteins in cancerous versus non-cancerous cells. The study posited that cancer cells are more prone to ER stress due to increased demands for protein synthesis and stressful environments. α-Mangostin (7.5 and 15 μM) selectively induced ER stress in fast-dividing prostate cancer cells, possibly due to its role as a cell cycle inhibitor. In vivo experiments with xenograft mice showed that α-mangostin inhibited tumor growth at doses of 35 and 70 mg/kg without affecting body weight. Johnson and colleagues demonstrated α-mangostin’s anti-cancer effects against prostate cancer cells both in vitro and in vivo [42]. α-Mangostin (20, 40, 60, and 80 μM) treatment significantly decreased cell viability in LNCaP, 22Rv1, DU145, and PC3 cells, with half maximal inhibitory concentration (IC_50_) values ranging from 5.9 to 22.5 μM. α-Mangostin inhibited clonogenic potential, induced cell cycle arrest, and promoted apoptosis in prostate cancer cells. Cell-free kinase assays revealed α-mangostin’s (10 μM) inhibition of cyclin/CDK proteins, particularly cyclin D1/CDK4. This inhibition was accompanied by an increase in p27^Kip1^ expression and a decrease in cyclins D1 and D3. Downstream effects included decreased phosphorylated retinoblastoma protein (p-Rb) and cyclin E expression. In vivo experiments in male athymic nude mice implanted with 22Rv1 cells demonstrated that α-mangostin (100 mg/kg) was well tolerated, with no significant difference in body weight compared with controls. Mice receiving α-mangostin (100 mg/kg) showed a significant delay in tumor formation and a 65% reduction in tumor volume compared with controls.

Kim and colleagues conducted an in vitro study to investigate the anti-cancer effects of α-mangostin in pancreatic cancer cell lines (MIA PaCa-2 and PANC-1) [43]. α-Mangostin treatment reduced cell viability and induced apoptosis in both cells, as evidenced by increased levels of cleaved caspase-3, cleaved PARP, and Bax. Additionally, α-mangostin induced autophagy in MIA PaCa-2 and PANC-1 cells, indicated by increased levels of LC3II and decreased levels of p62. Xu and colleagues investigated the mechanism of α-mangostin in pancreatic cancer cell lines (BxPc-3 and PANC-1), highlighting its inhibition of cell viability and epithelial-mesenchymal transition (EMT) by targeting the PI3K/Akt pathway [44]. α-Mangostin (2, 4, 6, 8, 16, and 32 μM) treatment significantly reduced the viability of pancreatic cancer cells in a time-dependent manner and induced apoptosis, increasing Annexin V-FITC and PI-positive cells, along with a dose-dependent reduction in anti-apoptotic Bcl-2 protein levels and an increase in cleaved caspase-3 levels. Additionally, α-mangostin (2, 4, 6, 8, 16, and 32 μM) caused cell cycle arrest at the G1/G0 phase by downregulating cyclin-D1, leading to reduced proliferation. In vivo tumor xenograft experiments in male BALB/c nude mice confirmed the inhibitory effect of α-mangostin (50 and 100 mg/kg) on pancreatic cancer growth, with significant reductions in tumor volume without adverse effects on the animals’ body weights. Ma and colleagues explored α-mangostin’s effect on pancreatic CSC cell lines (AsPC-1 and PANC-1) [45]. α-Mangostin (1–10 μM) inhibited cell proliferation in a dose-dependent manner, induced apoptosis, and inhibited the self-renewal capacity of CSCs. α-Mangostin (1–10 μM) also impaired spheroid and colony formation, downregulated CSC markers, and pluripotency-maintaining factors, suggesting its potential in targeting pancreatic CSC characteristics. Hafeez and colleagues explored α-mangostin’s anti-cancer effects on human pancreatic cancer cell lines (PANC-1, BxPC3, and PL-45) [14]. α-Mangostin (2, 4, 5, 6, 7.5, 10, 12, 15, 16, 20, 24, and 30 μM) inhibited cell viability in a dose-dependent manner, induced apoptosis, reduced intracellular ROS levels, suppressed proliferation markers, and inhibited invasion. α-Mangostin (7.5, 15, 20, and 30 μM) treatment reduced the activity and expression of matrix metalloproteinase-9 (MMP-9), while increasing tissue inhibitor of metalloproteinase-1 (TIMP-1) levels. α-Mangostin (7.5, 15, 20, and 30 μM) also inhibited cyclin D1 and interleukin-6 receptor glycoprotein 130 (gp130) expression, downstream targets of signal transducer and activator of transcription 3 (STAT3), and decreased Bcl-3 protein levels. α-Mangostin (7.5, 15, 20, and 30 μM) induced cell cycle arrest in the G0/G1 phase and inhibited the invasion and colony formation of pancreatic cancer cells in vitro. In vivo, α-mangostin (6 mg/kg) administration inhibited the growth of pancreatic cancer cell-derived orthotopic and ectopic xenograft tumors, reduced tumor volume and weight, and decreased proliferation markers without showing any toxicity [14].

In a combined study led by Hsieh and colleagues, the apoptotic-inducing potential of α-mangostin in human hepatocellular carcinoma (HCC) cells was explored both in vitro and in vivo [46]. α-Mangostin (10, 20, 30, and 40 μM) demonstrated dose- and time-dependent inhibition of SK-Hep-1, HA22T/VGH, Huh-7, PLC/PRF/5, and HepG2 cell growth, suppressing long-term colony formation and inducing chromatin condensation, nuclear fragmentation, and increased sub-G1 phase population, indicative of apoptosis. Treatment with α-mangostin (10, 20, 30, and 40 μM) also led to the activation of the caspase cascade, including the cleaved forms of caspase-3, caspase-6, caspase-7, caspase-8, caspase-9, and PARP, along with dissipation of mitochondrial membrane potential and release of cytochrome c into the cytosol. Additionally, α-mangostin (10, 20, 30, and 40 μM) downregulated Bcl-2-related proteins and upregulated Bak and Bax expression, suggesting the activation of the mitochondrial pathway in α-mangostin-induced apoptosis. In vivo experiments in xenograft BALB/c male mice (18–22 g, 5 weeks) further supported intraperitoneally administered α-mangostin’s (8 mg/kg) anti-tumor efficacy, significantly suppressing tumor growth without affecting body weight. Wudtiwai and colleagues showcased α-mangostin’s ability to sensitize anoikis in HepG2 [47]. Anoikis-resistance, a crucial step in cancer progression, involves alterations in cell survival, drug resistance, and EMT. α-Mangostin (1, 2, 5.5, 7, and 14 μM) also inhibited re-adhesion and migration, decreased MMP-2 and MMP-9 secretion, and reversed EMT phenotypes [47]. Moreover, Zhang and colleagues elucidated α-mangostin’s (1, 2, 5, 10, 20, and 40 μM) anti-cancer mechanism in HCC cell lines (HepG2, Huh-7, SK-Hep-1, and SMMC-7721) [48]. α-Mangostin (1, 2, 5, 10, 20, and 40 μM) inhibited HCC cell growth, clonogenic potential, induced G2 to mitosis (M) phase cell cycle arrest, and apoptosis in vitro, as evidenced by flow cytometry and morphological observations. Mechanistically, α-mangostin (5, 10, and 20 μM) inhibited signal transducer and activator of transcription 3 (STAT3) activation by suppressing its phosphorylation, dimerization, and nuclear translocation, resulting in decreased expression of STAT3-targeted genes involved in cell proliferation and survival. In vivo, α-mangostin (50 mg/kg) significantly inhibited the growth of HCC tumors in male BALB/c nude mice, accompanied by reduced antigen Ki-67 expression, decreased Bcl-2 expression, inhibited STAT3 phosphorylation, and induced Src homology region 2 domain-containing phosphatase-1 (SHP1) expression.

Markowicz and colleagues investigated the anti-cancer properties of α-mangostin on squamous cell carcinoma (SCC-15) and glioblastoma multiforme (U-118 MG) cells [49]. α-Mangostin (7.5, 10, 20, and 40 μM) exhibited concentration- and cell-type-dependent effects in both toxicity and proliferation assays. Squamous carcinoma cells showed the strongest response, with significant viability reduction at 7.5 μM concentration and total viability loss at higher concentrations. α-Mangostin (7.5, 10, 20, and 40 μM) induced apoptosis in all tested cells, with lower concentrations affecting cancer cells more than normal cells. Notably, SCC-15 and U-118 MG cells exhibited significant caspase activity increases at 10 μM, whereas fibroblasts required 20 μM. Indeed, the activity of caspase-3 and caspase-7 in squamous carcinoma cells was notably higher compared with glioma cells at 10 μM. Another study conducted an in vitro study examining the impact of α-mangostin on MMP-2 and MMP-9 expression in head and neck squamous cell carcinoma (HNSCC) cell lines (HN-22, HN-30, and HN-31) [50]. α-Mangostin (1.22, 2.44, and 4.88 μM) inhibited cell growth and decreased MMP-2 and MMP-9 expression at the transcription level. Another study explored the cytotoxic impact of α-mangostin (1–5 μg/mL) on head and neck squamous cell carcinoma (HNSCC) cells, revealing its dose- and time-dependent cytotoxicity and significant inhibition of cell proliferation observed at 3 μg/mL [51]. Morphological changes indicative of apoptosis were observed, accompanied by downregulation of anti-apoptotic Bcl-2 messenger ribonucleic acid (mRNA) and protein levels and upregulation of pro-apoptotic Bax mRNA and protein levels, along with increased expression of p53 mRNA and protein levels. Kwak and colleagues investigated α-mangostin’s potential as an anti-cancer agent against human oral squamous cell carcinoma (OSCC) cell lines (HSC-2, HSC-3, and HSC-4) [52]. α-Mangostin (1–10 μM) treatment reduced OSCC cell viability dose- and time-dependently, inducing apoptosis characterized by nuclear condensation and fragmentation, loss of mitochondrial membrane potential, and release of cytochrome c from mitochondria into the cytosol. α-Mangostin (8 μM) induced activation of the intrinsic apoptosis pathway, as evidenced by increased expression of the pro-apoptotic protein Bak and cleaved forms of caspase-3 and PARP, accompanied by G1 phase arrest and modulation of CDK/cyclin complex proteins involved in cell cycle progression and upregulation of p21, a CDK inhibitor.

Several studies have explored the potential anti-cancer effects of α-mangostin across different types of melanoma cells. Beninati and colleagues investigated α-mangostin’s impact on B16-F10 mouse melanoma cells as well as human SK-MEL-28 and A375 melanoma cells in vitro [53]. They found that α-mangostin treatment at concentrations of 5, 10, and 15 μM reduced cell proliferation without significant cytotoxicity. Flow cytometry analysis revealed an increase in the subG0/G1 cell population, particularly at 15 μM. Wang and colleagues delved into α-mangostin’s cytotoxic effects specifically on human melanoma SK-MEL-28 cells, uncovering its ability to induce apoptosis through both extrinsic and intrinsic pathways [54]. α-Mangostin treatment at concentrations of 5 μg/mL and 7.5 μg/mL increased caspase 8 and 9 activity, led to cytochrome c release from mitochondria, induced modulation of cell cycle-related genes, and downregulated Akt and NF-κB mRNA expression. Additionally, α-mangostin (5 and 7.5 μg/mL) decreased BRAF V600E mutant gene expression at the same concentrations. In another aspect, Zhou and colleagues explored α-mangostin’s effect on melanogenesis in mouse B16F10 cells [55]. They discovered that α-mangostin at concentrations of 3, 6, and 9 μM reduced melanin production by suppressing tyrosinase activity and downregulating key melanogenesis-related genes, including melanocyte-inducing transcription factor (MITF). Additionally, Wang and colleagues investigated α-mangostin’s therapeutic potential in treating skin tumorigenesis induced by 7,12-dimethylbenzanthracene/12-O-tetradecanoylphorbol 13-acetate (DMBA/TPA) in female ICR mice [56]. They found that α-mangostin treatment at doses of 5 and 20 mg/kg significantly inhibited tumor formation and growth, decreased tumor incidence rate and multiplicity, and promoted apoptosis and autophagy.

Yu and colleagues investigated the anti-cancer potential of α-mangostin against human ovarian cancer cells (OVACAR-3) [57]. α-Mangostin treatment at concentrations ranging from 0 to 200 μM demonstrated both dose- and time-dependent suppression of proliferation and clonogenic potential. Additionally, α-mangostin-induced apoptosis was characterized by morphological changes, increased expression of pro-apoptotic proteins (such as Bax, caspase-3, caspase-8, and caspase-9), and decreased expression of the anti-apoptotic protein Bcl-2. Notably, α-mangostin (5, 25, 50, 100, and 200 μM) also affected mitochondrial membrane potential and ROS production, contributing to its cytotoxic effect on OVACAR-3 cells. Furthermore, Ittiudomrak and colleagues explored α-mangostin’s interaction with the chemotherapeutic drug doxorubicin in ovarian adenocarcinoma cells (SKOV-3) [58]. They found that α-mangostin inhibited cell growth in a dose-dependent manner, induced significant necrosis, and caused cell cycle arrest at the G2/M phase. Additionally, α-mangostin modulated gene expression related to apoptosis and cell cycle regulation, suggesting its potential in combination therapy strategies for ovarian cancer.

Lee and colleagues investigated α-mangostin’s apoptotic effects on YD-15 tongue mucoepidermoid carcinoma cells both in vitro and in vivo [59]. Colorimetric assays showed that treatment with α-mangostin (10 and 15 µM) led to a concentration-dependent decrease in cell viability of YD-15 cells, accompanied by increased chromatin condensation indicative of apoptosis revealed after 4′,6-diamidino-2-phenylindole (DAPI) staining. α-Mangostin (10 and 15 µM) treatment also resulted in increased activity of caspase-3 and caspase-9, along with elevated levels of cleaved PARP, indicating caspase activation associated with apoptosis induction. In vivo studies demonstrated that α-mangostin (10 and 20 mg/kg) administration significantly inhibited tumor volume and weight in a dose-dependent manner, with increased apoptosis observed in α-mangostin-treated mice compared with controls. Another significant in vitro study by Krajarng and colleagues explored the anti-cancer effects of α-mangostin (5, 10, 15, 20, and 30 μg/mL) in human chondrosarcoma cells (SW1353) [60]. α-Mangostin (5, 10, 15, 20, and 30 μg/mL) treatment inhibited cell proliferation in a dose- and time-dependent manner, accompanied by nuclear condensation and fragmentation indicative of apoptosis. Activation of caspase-3, caspase-8, and caspase-9, along with alterations in Bcl-2 and Bax expression, further supported mitochondrial dysfunction and cytochrome c release. Additionally, α-mangostin (5, 10, 15, 20, and 30 μg/mL) downregulated ERK, JNK, and Akt signaling pathways, suggesting its role in modulating key regulators of cell proliferation and apoptosis in SW1353 cells. Rojas-Ochoa and colleagues investigated the pro-apoptotic effects of α-mangostin (10, 15, 20, and 40 μM) on human medulloblastoma cells (Daoy) [61]. α-Mangostin (10, 15, 20, and 40 μM) reduced cell viability in a dose-dependent manner and induced G2/M phase arrest and apoptosis, as evidenced by flow cytometry [61,62]. Treatment with α-mangostin (10, 15, 20, and 40 μM) induced oxidative stress, as indicated by decreased glutathione levels, increased levels of oxidized glutathione (GSSG), and elevated carbonyl protein levels.

In a comprehensive study conducted by Aukkanimart and colleagues, the cytotoxic effects of α-mangostin were explored both in vitro and in vivo, focusing on its impact on white blood cells and human M214 cholangiocarcinoma (CCA) cell lines (KKU-M214 and Ham-1) [63]. Interestingly, α-mangostin (1.5, 4, 30, and 60 μg/mL) did not affect white blood cells at various concentrations, but it significantly reduced the growth of CCA cell lines at 1.36 μg/mL, inducing morphological changes characteristic of apoptosis such as membrane blebbing, cell shrinkage, and nuclear condensation and fragmentation. Moreover, α-mangostin (1 and 1.5 μg/mL) treatment led to cell cycle arrest at the G1 phase and stimulated apoptosis in CCA cell lines. α-Mangostin (1, 1.5, 2, 4, and 8 μg/mL) treatment increased the expression of caspase-3, p53, and Bax in a dose- and time-dependent manner, while Bcl-2 expression remained unaffected. In a hamster model, α-mangostin (100 mg/kg) significantly reduced tumor size, bile duct proliferation, and the expression of proliferating cell nuclear antigen (PCNA) in tumor tissue. Chao and colleagues investigated α-mangostin’s anti-cancer effects in human glioblastoma cell lines (GBM8401 and DBTRG-05MG), revealing a different mechanism of action [64]. α-Mangostin (2.5, 5, 7.5, and 10 μM) decreased cell viability and clonogenic survival in a concentration-dependent manner without inducing apoptosis. Instead, α-mangostin (2.5, 5, 7.5, and 10 μM) induced autophagy in both cell lines, as shown by the appearance of autophagic vacuoles, increased monodansylcadaverine (MDC) accumulation, and redistribution of LC3. Knockdown of Beclin1 or inhibition of autophagy decreased α-mangostin-induced cytotoxicity, indicating autophagy as the major mechanism of cell death. Cells deficient in AMP-activated protein kinase (AMPK) showed reduced autophagy in response to α-mangostin (10 μM) treatment. In vivo experiments in male nude BALB/cA-ν (*ν*/*ν*) mice demonstrated that α-mangostin (2 mg/kg) inhibited tumor growth in mice without signs of toxicity. TEM analysis showed an increase in autophagocytic vacuoles in tumors of α-mangostin-treated mice. Furthermore, increased phosphorylation of AMPK and its target regulatory-associated protein of mTOR (RAPTOR) was observed in tumors from α-mangostin-treated mice compared with vehicle-treated mice.

In an in vitro study, the cell death effects of α-mangostin on PC12 rat pheochromocytoma cells were investigated [65]. α-Mangostin had the highest potency at a half maximal effective concentration (EC_50_) value of 4 μM. α-Mangostin (30 μM) induced typical apoptotic DNA fragmentation and caspase-3 cleavage, indicative of apoptosis. Flow cytometric analysis showed α-mangostin (1–50 μM) induced apoptosis in a concentration- and time-dependent manner. α-Mangostin (30 μM) depolarized mitochondria and led to cytochrome c release, indicating involvement of the mitochondrial apoptotic pathway. No significant caspase-8 activity was observed, suggesting the death receptor pathway might not be involved. α-Mangostin (30 μM) inhibited Ca^2+^-ATPase activity, correlating with its apoptotic effects. Furthermore, α-mangostin (30 μM) phosphorylated and activated JNK, suggesting involvement of ER stress-dependent signaling in α-mangostin-induced apoptosis.

**Figure 1:**
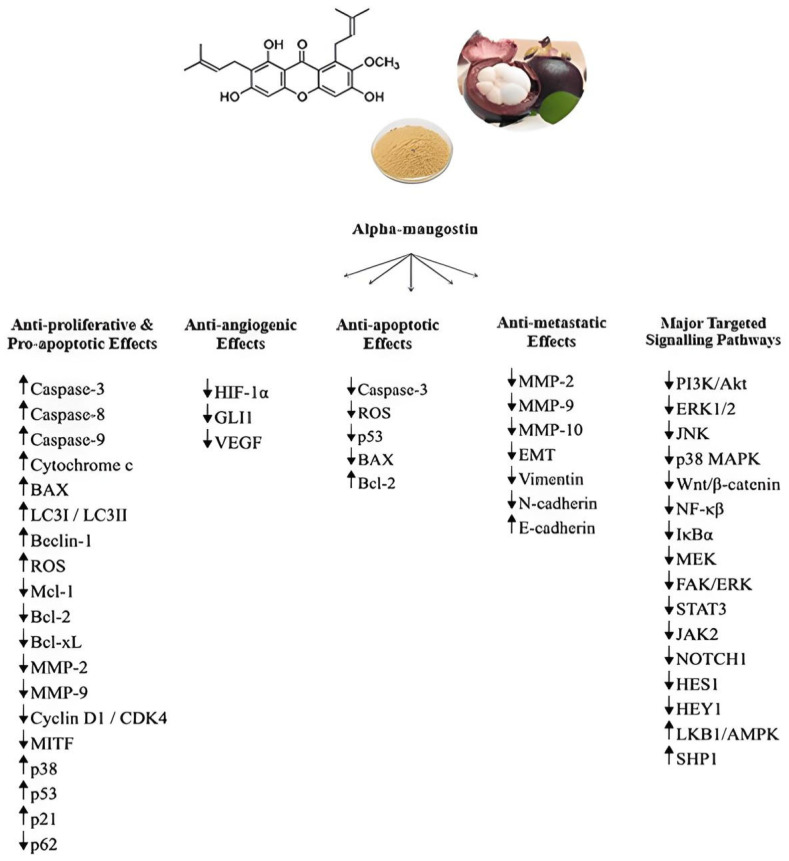
A schematic representation of the major molecular and cellular mechanisms and targeted signaling pathways underlying the anti-angiogenic, anti-metastatic, pro-apoptotic/anti-proliferative, and anti-apoptotic activities of α-mangostin. “↑” means upregulation, while “↓” means downregulation.

Khan and colleagues identified α-mangostin as a promising inhibitor of microtubule affinity-regulating kinase 4 (MARK4), which is overexpressed in various cancers [66]. α-Mangostin (1–50 μM) inhibited MARK4 kinase activity, leading to decreased cell proliferation, induction of apoptosis, and G0/G1 cell cycle arrest in cancer cells. Additionally, α-mangostin (1–50 μM) treatment reduced tubulin-associated unit (tau) phosphorylation, consistent with MARK4 inhibition (Figure 1).

These studies collectively shed light on the diverse mechanisms through which α-mangostin exerts its anti-cancer effects, ranging from apoptosis induction and inhibition of key metabolic pathways to modulation of cellular stress responses and alterations in tumor microenvironments. α-Mangostin’s multifaceted approach makes it a promising candidate for further exploration as a potential anti-cancer agent across various cancer types.

## 3. Anti-Metastatic Effects of α-Mangostin

α-Mangostin exhibits promising anti-metastatic properties, as evidenced by various in vitro studies across different cancer types. Chen and colleagues investigated the effects of α-mangostins (2, 4, 8, and 12 μM) on human renal carcinoma cell metastasis, revealing significant inhibition of migration and invasion in CaKi-1, ACHN, A-498, 786-O, and HK-2 cells in a concentration-dependent manner [9]. Interestingly, α-mangostin (2, 4, 8, and 12 μM) did not affect normal human proximal tubule epithelial cells, indicating its selectivity towards cancer cells. Moreover, α-mangostin (7.5, 10, 20, and 40 μM) treatment was found to reduce cell motility and adhesion in squamous carcinoma cells, glioma, and fibroblast cells, indicating its potential to impede cancer metastasis [19,49]. Similarly, Beninati and colleagues demonstrated that α-mangostin (5, 10, and 15 μM) reduced B16-F10 cells’ adhesion to basement proteins, as well as the plasticity and invasive potential of the melanoma cells, dose-dependently [53]. Additionally, α-mangostin (5, 10, and 15 μM) decreased MMP-9 activity, indicating reduced extracellular matrix degradation. Furthermore, it was found that α-mangostin (12.5 and 29.98 μM) treatment reduced changes in gene expression associated with metastasis-related processes in ovarian cancer cells [67]. However, there were no significant changes in certain factors like CDH6, CDH11, FAT1, integrin alpha 7 (ITGA7), and spleen-associated tyrosine kinase (Syk). Treatment with α-mangostin (12.5 and 29.98 µM) reduced increases in MMP-2 and MMP-10 expression from exosomes from untreated cancer cells. Exosomes from SKOV-3 and TOV-21G ovarian cancer cells increased the expression of mouse double minute 2 homolog (MDM2) and interleukin 18 (IL-18) in fibroblasts but were reduced after α-mangostin (12.5 and 29.98 µM) treatment on gene expression in fibroblasts [67]. This suggests that α-mangostin may not only directly affect tumor cells but also modulate metastasis through exosome-mediated mechanisms.

In addition to its effects on cell motility and invasion, α-mangostin has been shown to inhibit EMT, a critical step in metastasis, in pancreatic cancer cells, as evidenced by increased epithelial (E)-cadherin levels and decreased vimentin and N-cadherin levels in both protein and mRNA expression [44]. This inhibition is associated with the downregulation of the PI3K/Akt pathway, highlighting the multifaceted anti-metastatic mechanisms of α-mangostin. Another in vitro study aimed to explore the anti-metastatic properties of α-mangostin (1, 2.5, 5, 7.5, 10, 12.5, 15, and 17.5 μM) on phorbol myristate acetate (PMA)-induced MMP-2 and MMP-9 expressions in A549 and WI-38 lung adenocarcinoma cells [68]. α-Mangostin (5 μM) inhibited PMA-induced cell adhesion, invasion, and migration of A549 cells through activation of the αvβ3 integrin receptor. Gelatin zymography and reverse transcription polymerase chain reaction (RT-PCR) demonstrated that α-mangostin (1, 2.5, and 5 μM) reduced MMP-2 and MMP-9 activities and mRNA expressions, respectively, in response to PMA. Similarly, Lee and colleagues reported that α-mangostin (2, 4, and 6 μM) inhibited TPA-induced adhesion, invasion, and migration of MCF-7 breast adenocarcinoma cells in a dose-dependent manner, with 6 μM significantly reducing these properties [69]. α-Mangostin (2, 4, and 6 μM) also suppressed MMP-2 and MMP-9 activities, protein expressions, and mRNA expressions induced by TPA.

Yuan and colleagues showed that α-mangostin (5, 7.5, 10 and 15 μM) reduced cell proliferation in a dose- and time-dependent manner without significant cytotoxicity at concentrations ≤ 5 μM [70]. Subsequent wound healing and invasion assays demonstrated that α-mangostin (5 μM) inhibited migration and invasion of pancreatic cancer cells induced by lipopolysaccharide (LPS). Moreover, α-mangostin (5 μM) suppressed ERK1/2 phosphorylation, and this effect was enhanced by ERK small interfering RNA (siRNA) transfection, leading to decreased expression of MMP-9 and MMP-2 and increased expression of E-cadherin [70]. Wang and colleagues studied the anti-invasive effects of α-mangostin on human melanoma SK-MEL-28 and squamous cell carcinoma A-431 cells [71]. α-Mangostin (0.5–2.5 μg/mL for SK-MEL-28 and 0.5–1.25 μg/mL for A-431) inhibited cell motility, migration, invasion, and adhesion in both cells in a concentration-dependent manner. It downregulated the expression of metastasis-related gene products, including MMP-2, MMP-9, NF-κB, and Akt in A-431 cells, and MMP-2, NF-κB, and IκBα in SK-MEL-28 cells as observed through quantitative reverse transcription (qRT)-PCR analysis.

Moreover, α-mangostin treatment led to a dose-dependent reduction in cell migration and invasion potential across various cancer cells, including pancreatic, lung, and ovarian cancer cells [23,26,57,66]. Notably, α-mangostin’s anti-metastatic effects were also observed in vivo in a murine model of mammary tumors, where α-mangostin (20 mg/kg/day) treatment reduced metastatic expansion and lymph node involvement [17]. Additionally, α-mangostin (20 mg/kg/day) treatment reduced the number of dilated lymphatic vessels containing intraluminal tumor cells, suggesting a decrease in tumor cell migration into lymphatic vessels (Figure 1). Overall, these findings suggest that α-mangostin holds promise as a potential anti-metastatic agent through its ability to inhibit cancer cell migration, invasion, and EMT, as well as modulate gene expression associated with metastasis. Further research is warranted to elucidate the precise mechanisms underlying α-mangostin’s anti-metastatic effects and its potential clinical applications in cancer therapy.

## 4. Anti-Angiogenic Effects of α-Mangostin

α-Mangostin has emerged as a promising candidate for inhibiting angiogenesis, a critical process implicated in tumor progression and metastasis. Lei and colleagues demonstrated that α-mangostin, at a concentration of 16 μM, exerted multifaceted effects on PSCs and pancreatic cancer invasion [10]. Notably, α-mangostin inhibited hypoxia-inducible factor 1a (HIF-1α) accumulation, suppressed glioma-associated oncogene homolog 1 (GLI1) expression under hypoxic conditions, and hindered PSC activation, collectively impeding the invasive potential of pancreatic cancer cells. The pivotal role of GLI1 in mediating the anti-tumor effects of α-mangostin was further underscored by the attenuation of a-smooth muscle actin (α-SMA) expression in PSCs upon GLI1 knockdown, highlighting the intricate molecular mechanisms underlying α-mangostin’s anti-angiogenic properties.

In a study by Jittiporn and colleagues, the impact of α-mangostin on angiogenesis was investigated using bovine retinal endothelial cells (REC) [72]. Initial experiments established a non-toxic dose range of α-mangostin (1–8 μM) for REC treatment. Subsequent analysis revealed dose-dependent inhibition of hypoxia-induced ROS formation and vascular endothelial growth factor (VEGF)-induced permeability by α-mangostin (1, 4, and 8 μM). Moreover, α-mangostin exhibited dose-dependent attenuation of VEGF-induced proliferation, migration, cellular alignment, and vascular sprouting in both in vitro and ex vivo assays. Mechanistically, α-mangostin (1, 4, and 8 μM) suppressed VEGF receptor 2 (VEGFR2) and ERK1/2-MAPK signaling in REC treated with VEGF, elucidating its inhibitory effect on key angiogenic pathways (Figure 1). Collectively, these findings underscore the potential of α-mangostin as a potent inhibitor of angiogenesis, offering insights into its therapeutic utility for mitigating tumor angiogenesis and impeding cancer progression.

## 5. Antioxidant Effects of α-Mangostin

α-Mangostin has garnered attention not only for its anti-cancer properties but also for its potential as an antioxidant agent. Initially, investigations into its antioxidant effects yielded mixed results. Itoh and colleagues found that α-mangostin treatment at 20 μM did not reduce intracellular ROS levels nor exhibit significant radical-scavenging activity in RBL-2H3 cells [11]. However, subsequent studies shed light on its antioxidative potential. Ibrahim and colleagues discovered that α-mangostin exhibited selective cytotoxicity against cancer cells while sparing normal cells, even at concentrations as high as 30 µg/mL [13]. This finding hinted at the compound’s ability to target cancer-specific pathways while leaving healthy cells unharmed. Further research delved into the mechanisms underlying α-mangostin’s antioxidant effects. Khan and colleagues demonstrated that α-mangostin treatment, ranging from 1 to 50 μM, effectively reduced ROS levels in MCF-7 and HepG2 cells, indicating its antioxidant properties [66]. This reduction in ROS levels suggested that α-mangostin could potentially alleviate oxidative stress, particularly in conditions associated with elevated ROS production. Moreover, α-mangostin was found to modulate antioxidant enzyme activity in a dose-dependent manner. Zhang and colleagues observed that treatment with α-mangostin at concentrations ranging from 1 to 10 μM enhanced antioxidant enzyme activity at lower doses but resulted in decreased activity at IC_50_ concentrations [24]. This biphasic modulation suggests a nuanced interplay between α-mangostin concentration and antioxidant response. The involvement of ROS in α-mangostin-mediated cytotoxicity was underscored by the findings of Zhang and colleagues [24]. Co-treatment with the antioxidant N-acetylcysteine (NAC) not only reduced ROS generation but also restored cell viability in α-mangostin-treated A549 cells, particularly implicating ROS in α-mangostin-induced cytotoxicity in NSCLC cells.

In summary, while early studies may have cast doubt on α-mangostin’s antioxidant properties, subsequent research has highlighted its ability to mitigate ROS levels and modulate antioxidant enzyme activity. These findings underscore the compound’s potential not only as an anti-cancer agent but also as a promising antioxidant for combating oxidative stress-related disorders.

## 6. Signaling Pathways Underlying the Anti-Cancer Effects of α-Mangostin

α-Mangostin exerts potent anti-cancer effects through modulation of various signaling pathways implicated in cell proliferation, apoptosis, metastasis, and tumor progression. Studies have elucidated the intricate molecular mechanisms by which α-mangostin interferes with key signaling cascades to inhibit cancer cell growth and promote apoptosis. In vitro investigations by Itoh and colleagues revealed that α-mangostin (20 μM) significantly suppressed the phosphorylation of Syk, phospholipase C gamma 1 (PLCγ1), PLCγ2, and ERK1/2, implicating its role in suppressing degranulation [11]. α-Mangostin (20 μM) also inhibited the PI3K/Akt signaling pathway, potentially activating the pro-apoptotic protein Bax [7,47]. Furthermore, α-mangostin (10, 20, 30, 40, and 50 μM) demonstrated suppression of the Wnt/β-catenin pathway in osteosarcoma cells by reducing the protein levels of Wnt3a, phosphorylated glycogen synthase kinase 3 beta (GSK3β), and nuclear β-catenin [25].

In SW1353 cells, α-mangostin downregulated total and phosphorylated ERK and JNK and decreased phosphorylated Akt, thereby inhibiting cell proliferation and inducing apoptosis [40,60,73]. Hung and colleagues [73] and Lee and colleagues [69] also found that α-mangostin (1, 3, and 5 μg/mL) reduced the nuclear levels of NF-κB, c-Fos, and c-Jun, transcription factors involved in MMP and urokinase-type plasminogen activator (u-PA) regulation, and inhibited AP-1 DNA binding activity. Similarly, α-mangostin inhibited RCC cell migration and invasion by suppressing the activation of MEK and ERK without affecting JNK and p38 phosphorylation [9]. In another study, however, Zhou and colleagues reported that α-mangostin (3, 6, and 9 μM) upregulated the phosphorylation of GSK3β and ERK, suggesting their involvement in the melanin downregulation process in B16F10 cells [55].

Treatment with α-mangostin also led to inhibition of ERK1/2 and p38 activation, along with decreased c-Myc expression in YD-15 cells. Upon α-mangostin (10 and 15 µM) treatment, tumor tissue from mice with YD-15 tumor xenografts showed reduced phosphorylated levels of ERK1/2 and p38. In vivo studies demonstrated that α-mangostin (10 and 20 mg/kg) administration significantly inhibited tumor volume and weight in a dose-dependent manner. Additionally, immunohistochemical analysis revealed decreased expression of Ki-67 in α-mangostin-treated mice, indicating inhibition of tumor growth through induction of apoptosis in YD-15 cells, as further evidenced through DNA fragmentation assays [59,74]. α-Mangostin (1, 2.5, and 5 μM) inhibited focal adhesion kinase (FAK) and ERK phosphorylation induced by PMA, implicating the FAK/ERK signaling pathway in its mechanism of action [68,74]. Further experiments using FAK and ERK siRNA confirmed that α-mangostin’s (5 μM) inhibition of MMP-2 and MMP-9 expressions, invasion, and migration occurred mainly through the FAK/ERK pathway. Treatment with α-mangostin (5 μM) inhibited inhibitors of nuclear factor kappa B alpha (IkBα) degradation and p50/p65 nuclear translocation induced by PMA. This inhibition was accompanied by reduced NF-κB DNA binding activity [68].

Additionally, α-mangostin (7.5, 15, and 30 μM) treatment influenced the MAPK pathway in T47D cells, leading to increased phosphorylation of p38 and JNK1/2, while phosphorylation of ERK1/2 and c-Raf was decreased, contributing to apoptosis induction [12,26]. Treatment of T47D cells with 30 μM α-mangostin led to decreased HER2 phosphorylation at Tyr^1221/1222^, resulting in the inactivation of RAS/Raf1/MEK/ERK and PI3K/Akt transduction cascades. This inhibition of PI3K/Akt signaling cascade by α-mangostin (7.5, 15, and 30 μM) was further evidenced by suppressed Akt phosphorylation at Ser^473^ and Thr^308^, leading to inhibition of cell proliferation and induction of apoptosis. Additionally, α-mangostin (7.5, 15, and 30 μM) decreased phosphorylation of ERα at Ser^104/106^ and Ser^118^ at 6 h, suggesting its ability to suppress ERα phosphorylation, thereby inhibiting cell proliferation and survival in T47D cells [12]. Similarly, Kim and colleagues [43] demonstrated that α-mangostin induced autophagy through the AMPK/mammalian target of rapamycin (mTOR) and p38 pathways, as evidenced by increased phosphorylation of AMPK and p38 and decreased phosphorylation of mTOR at earlier time points [57].

α-Mangostin (5, 10, and 20 μg/mL) exhibited significant inhibitory effects on the NF-κB induced by TNFα, thereby suppressing NF-κB activation and its nuclear translocation [13,14]. In gastric adenocarcinoma cells, α-mangostin-induced apoptosis was associated with mitochondrial dysfunction and inhibition of the STAT3 signaling pathway. Immunofluorescence and Western blot analysis demonstrated reduced phosphorylated STAT3 (pSTAT3) levels in treated cells compared with controls after 24 h, suggesting involvement of the STAT3 signaling pathway in α-mangostin-induced apoptosis. SGC-7901 and BGC-823 cells treated with α-mangostin (7 μg/mL) showed significant reductions in the levels of the anti-apoptotic proteins Bcl-xL and Mcl-1, which are targets for STAT3. This reduction correlates with the inhibition of STAT3 phosphorylation and activity by α-mangostin (7 μg/mL), suggesting that α-mangostin (7 μg/mL) induces cell growth inhibition and apoptosis by inhibiting STAT3-dependent induction of Bcl-xL and Mcl-1 [31]. Additionally, α-mangostin (10, 15, 20, 25, and 30 μM) treatment reduced Epstein-Barr virus-induced 3 (EBI3) and phosphorylated STAT3 levels, suggesting inhibition of the EBI3/STAT3 pathway. Overexpression of EBI3 attenuated α-mangostin-induced apoptosis and inhibition rates in SGC7901/CDDP cells, accompanied by altered autophagy-related protein expression. EBI3 overexpression also increased p-STAT3 levels and the p-STAT3/STAT3 ratio. These findings demonstrate that α-mangostin (10, 15, 20, 25, and 30 μM) facilitates CDDP-induced autophagy in SGC7901/CDDP cells and inhibits the EBI3/STAT3 pathway, contributing to enhanced chemosensitivity [32]. Additionally, α-mangostin (5, 10, and 20 μM) suppressed the activation of upstream kinases involved in STAT3 phosphorylation, including janus kinase 2 (JAK2), Src, ERK, and Akt. Notably, α-mangostin (5, 10, and 20 μM) increased the protein level of SHP1, which negatively regulates STAT3 signaling, and stabilized SHP1 protein by inhibiting its degradation via the ubiquitin-proteasome pathway [48] (Figure 1).

Chao and colleagues demonstrated that α-mangostin (10 μM) activated the liver kinase B1 (LKB1)/AMPK signaling pathway, leading to inhibition of mTOR complex 1 (mTORC1) activity and downstream targets such as p70S6K and 4E-BP1 [64]. The study confirmed the central role of AMPK in α-mangostin-induced autophagy and mTORC1 inhibition by establishing stable colonies lacking AMPK in GBM8401 and DBTRG-05MG cells. Furthermore, α-mangostin (1–10 µM) suppressed the Sonic hedgehog (Shh) signaling pathway and the transcription and expression of GLI, a component of the Shh pathway [45]. Moreover, in SW620 and HT29 cells, α-mangostin (2.5, 5, 10, 20, and 40 μM in SW260 and 0.25, 0.5, 1, and 2 μM in HT29) treatment downregulated the Notch signaling pathway components, including Notch1, Hes1, and Hey1, contributing to its anti-cancer effects [36]. These findings collectively highlight the diverse mechanisms by which α-mangostin exerts its anti-cancer effects through the modulation of multiple signaling pathways, providing insights into its potential as a therapeutic agent for cancer treatment.

Figure 1 provides a schematic representation of the known molecular and cellular mechanisms as well as the major targeted signaling pathways underlying the reported anti-cancer effects of α-mangostin.

Table 1 presents a detailed summary of the reported anti-cancer activities of α-mangostin, the dosages, the dosage regimens, and the experimental models, as well as whether the reported effects are dose-dependent and/or time-dependent.

## 7. Conclusions

The evidence presented in this review underscores the promising potential of α-mangostin as a natural anti-cancer agent. Through a comprehensive examination of in vitro and in vivo studies, amangostin has demonstrated remarkable cytotoxicity against various cancer types, including colon, glioblastoma, melanoma, oral squamous cell carcinoma, pancreatic cancer, and more. Its ability to selectively target cancer cells while sparing normal cells highlights its promising therapeutic profile. The multifaceted mechanisms underlying α-mangostin’s anti-cancer effects have been elucidated, revealing its capacity to induce apoptosis through modulation of intrinsic and extrinsic apoptotic pathways, mitochondrial dysfunction, and activation of caspases. Moreover, α-mangostin exerts regulatory effects on cellular processes such as autophagy, ER stress, and oxidative stress, further contributing to its anti-cancer efficacy. Preclinical studies have demonstrated the therapeutic potential of α-mangostin in xenograft and orthotopic tumor models, highlighting its efficacy and safety in vivo. Additionally, α-mangostin exhibits synergistic effects with conventional chemotherapeutic agents, suggesting its utility in combination therapies. Overall, α-mangostin emerges as a promising candidate for further development as a novel anti-cancer therapy. However, additional preclinical studies and clinical investigations are warranted to fully elucidate its clinical utility, optimize dosage regimens, and evaluate its efficacy in diverse patient populations. With continued research and development efforts, α-mangostin holds significant promise for advancing the field of cancer therapeutics and improving patient outcomes.

Despite its promising anti-cancer properties, α-mangostin has several limitations that hinder its clinical application. One major challenge is its poor water solubility and low bioavailability, which reduces its efficacy when administered in vivo. These pharmacokinetic issues complicate its absorption and distribution in the body, limiting its therapeutic potential. There is also a need for more comprehensive studies on its potential side effects, interactions with conventional chemotherapeutic drugs, and long-term safety. Addressing these limitations through advanced drug delivery systems or combination therapies may be crucial for the future use of α-mangostin as an effective anti-cancer agent.

## Figures and Tables

**Table 1:** A detailed summary of the main findings related to the anti-cancer properties of α-mangostin, the dosages, the dosage regimens, the experimental models, as well as whether the reported effects are dose-dependent and/or time-dependent.

Main Effects	Experimental Model	Dosage	Administration Mode and Duration	Dose-Dependent and/or Time-Dependent	References
α-Mangostin did not reduce intracellular ROS levels. α-Mangostin suppressed the phosphorylation of Syk, PLCγ1, PLCγ2, ERK1/2, and p-Akt.	RBL-2H3 cells	20 μM	Incubation (30 min)	Dose-dependent	[11]
α-Mangostin induced mitochondria-mediated apoptosis and caused G1-phase arrest. α-Mangostin upregulated p21cip1 expression while downregulating various proteins involved in cell cycle progression. α-Mangostin increased caspase-3, caspase-8, and caspase-9 activity. α-Mangostin reduced cytochrome c levels in mitochondrial fractions. α-Mangostin inhibited the PI3K/Akt signaling pathway.	MDA-MB23 cells	20 μM	Incubation (24 h)	Dose-dependent	[7]
α-Mangostin enhanced caspase-3, caspase-8, and caspase-9 expression. α-Mangostin decreased Bcl-2 and increased Bax expression. α-Mangostin downregulated total and phosphorylated ERK and JNK. α-Mangostin decreased phosphorylated Akt.	SW1353 cells	5, 10, 15, 20, and 30 μg/mL	Incubation (3, 6, 9, 12, and 24 h)	Dose-dependent and time-dependent	[60]
α-Mangostin induced cytotoxicity, damaging intracellular structures and increasing oxidative stress and elevated intracellular ROS.	A549 cells	2, 4, 6, 8, and 10 μg/mL	Incubation (24 h)	Dose-dependent	[22]
α-Mangostin induced apoptosis and increases ROS production. α-Mangostin activated caspase-3 and caspase-8 cascade. α-Mangostin upregulated CHOP and ATF6 expression. α-Mangostin suppressed Wnt/β-catenin pathway by reducing Wnt3a, p-GSK3β, and nuclear β-catenin.	143B and Saos-2 cells	10, 20, 30, 40, and 50 μM	Incubation (24 h and 48 h)	Dose-dependent and time-dependent	[25]
α-Mangostin suppressed tumor volume and weight.	Female athymic BALB/c nude mice (18–20 g, 5 weeks)	5 and 20 mg/kg	Intraperitoneal injection	Dose-dependent and time-dependent	
α-Mangostin inhibited the migration and invasion. α-Mangostin reduced MMP-9 expression and enzyme activity. α-Mangostin induced decreases in the phosphorylation of MEK and ERK.	CaKi-1, ACHN, A-498, 786-O, and HK-2 cells	2, 4, 8, and 12 µM	Incubation (24 h and 48 h)	Dose-dependent	[9]
α-Mangostin activated apoptotic markers, such as the cleaved forms of caspase-3, caspase-9, and PARP. α-Mangostin disrupted mitochondrial membrane potential. α-Mangostin increased Bax and cytochrome c expression and decreased Bcl-2 expression. α-Mangostin increased phosphorylation of p38 and enhanced ROS production.	SiHa and HeLa cells	10, 20, and 30 μM	Incubation (24 h and 48 h)	Dose-dependent	[28]
α-Mangostin inhibited tumor growth and reduced tumor size, volume, and weight without affecting body weight.	Female nude mice (BALB/c nu/nu) (5 weeks)	20 and 40 mg/kg	Intraperitoneal injection (3 times/week)	Dose-dependent	
α-Mangostin increased chromatin condensation and sub-G1 phase cells. α-Mangostin increased expression Bax and decreased expression of Bcl-2. α-Mangostin increased activity of caspase-3 and caspase-9, along with elevated levels of cleaved PARP. α-Mangostin inhibition of ERK1/2 and p38 activation, along with decreased c-Myc expression.	YD-15 cells	10 and 15 µM	Incubation (24 h)	Dose-dependent	[39]
α-Mangostin inhibited tumor volume and weight. α-Mangostin decreased expression of Ki-67.	Male BALB/c nude (nu/nu) mice (5 weeks)	10 and 20 mg/kg	Intraperitoneal injection (5 times/week)	Dose-dependent	
α-Mangostin inhibited colony formation and increased Bax oligomers. α-Mangostin reduced Mcl-1 expression and increased Bax/Bcl-2 ratio. α-Mangostin induced the activity of caspase-3, caspase-9, and PARP. α-Mangostin increased phosphorylation of p38 and JNK1/2. α-Mangostin decreased phosphorylation of ERK1/2 and c-Raf. α-Mangostin decreased HER2 phosphorylation at Tyr^1221/1222^. α-Mangostin suppressed Akt phosphorylation at Ser^473^ and Thr^308^. α-Mangostin decreased phosphorylation of ERα at Ser^104/106^ and Ser^118^.	T47D cells	7.5, 15, and 30 μM	Incubation (3, 6, 9, 12, and 24 h)	Dose-dependent and time-dependent	[12]
α-Mangostin increased both early and late apoptotic cells and reduced MMP. α-Mangostin increased cytochrome c release and upregulated the expression of Bax and downregulated Bcl-2. α-Mangostin induced activation of caspase enzymes, including caspase-3, caspase-7, caspase-8, and caspase-9. α-Mangostin decreased PCNA expression and induced PARP cleavage. α-Mangostin increased intracellular ROS levels. α-Mangostin induced an arrest of the cell cycle in the G0/G1 phase. α-Mangostin reduced nuclear translocation of NF-κB p65.	MCF-7 cells	5, 10, and 20 μg/mL	Incubation (24 h)	Dose-dependent	[13]
α-Mangostin reduced tumor volume.	Female Sprague–Dawley rats with rat LA7 mammary adenocarcinoma cells	30 and 60 mg/kg/day	Oral administration (2 times/week for 28 days)	Dose-dependent	
α-Mangostin reduced pSTAT3, Bcl-xL, and Mcl-1 levels.	BGC-823 and SGC-7901 cells	3, 5, 7, and 10 μg/mL	Incubation (6, 12, 18, 24, and 48 h)	Dose-dependent and time-dependent	[31]
α-Mangostin decreased adhesion and increased caspase activity.	U-118 MG and SCC-15 cells	7.5, 10, 20, and 40 μM	Incubation (48 h and 72 h)	Dose-dependent	[49]
α-Mangostin increased percentage of cells arrested in the subG-1 phase. α-Mangostin enhanced the activity of caspase-3, caspase-8, and caspase-9. α-Mangostin increased p-p53, Bax and Bmf, and the release of cytochrome c from the mitochondria to the cytosol. α-Mangostin depolarized mitochondria membrane and upregulated Bid, t-Bid, and FasR.	COLO 205, MIP-101, and SW620 cells	10, 20, 30, and 40 μg/mL	Incubation (0, 3, 6, 9, and 12 h)	Dose-dependent and time-dependent	[33]
α-Mangostin reduced cell proliferation and increased cleaved caspase-3 levels. α-Mangostin increased expression of ER stress markers and reduced PERK levels.	22Rv1 and LNCaP cells	7.5 and 15 μM	Incubation (24 h)	Dose-dependent	[74]
α-Mangostin inhibited tumor growth.	Athymic nude mice (22Rv1 xenograft model)	35 and 70 mg/kg	Intraperitoneal injection (2 times/week)	Dose-dependent	
α-Mangostin induced chromatin condensation, nuclear fragmentation, and increased the proportion of cells in the sub-G1 phase. α-Mangostin increased levels of cleaved active forms of caspase-3, caspase-6, caspase-7, caspase-8, caspase-9, and PARP. α-Mangostin induced dissipation of mitochondrial membrane potential and release of cytochrome c. α-Mangostin downregulated Bcl-2-related proteins and upregulated Bak and Bax expression. α-Mangostin decreased phosphorylation levels of p38 MAPK.	HA22T/VGH, SK-Hep-1, Huh-7, PLC/PRF/5, and HepG2 cells	10, 20, 30, and 40 μM	Incubation (24 and 48 h)	Dose-dependent and time-dependent	[46]
α-Mangostin suppressed tumor growth.	Male BALB/c mice (18–22 g, 5 weeks)	8 mg/kg	Intraperitoneal injection (3 times/week)	Dose-dependent and time-dependent	
α-Mangostin increased levels of cleaved caspase-3, cleaved PARP, and Bax. α-Mangostin induced autophagy through the AMPK/mTOR and p38 pathways. α-Mangostin increased levels of LC3II and decreased levels of p62. α-Mangostin increased phosphorylation of AMPK and p38 and decreased phosphorylation of mTOR.	MIA PaCa-2 and PANC-1 cells	1, 5, 10, and 20 μM	Incubation (48 h)	Dose-dependent	[43]
α-Mangostin enhanced the activity of caspase-9 and PARP-1. α-Mangostin suppressed the PI3K/Akt signaling pathway and inactivated MAPK pathways.	DLD-1, SW480, and COLO201 cells	5, 10, and 20 μM	Incubation (24, 48, and 72 h)	Dose-dependent and time-dependent	[34]
α-Mangostin increased the subG0/G1 cell population. α-Mangostin increased cell aggregation. α-Mangostin reduced adhesion to basement membrane proteins. α-Mangostin reduced plasticity and invasive potential of the melanoma cells. α-Mangostin decreased MMP-9 activity.	B16-F10, SK-MEL-28, and A375 cells	5, 10, and 15 μM	Incubation (24 and 48 h)	Dose-dependent	[53]
α-Mangostin induced G1-phase cell cycle arrest. α-Mangostin upregulated p27 levels and downregulated cyclins.	DLD-1 cells	20 μM	Incubation (24, 48, 72, and 96 h)	Dose-dependent	[35]
α-Mangostin upregulated endogenous MOAP-1 and downregulated Bcl-xL. α-Mangostin promoted the interaction of activated Bax with MOAP-1.	MCF-7 and MCF-7-CR cells	10, 20, and 30 μM	Incubation (12 and 48 h)	Dose-dependent and time-dependent	[8]
α-Mangostin increased expression of pro-apoptotic proteins and decreased expression of anti-apoptotic proteins. α-Mangostin increased levels of autophagy-related proteins LC3-II/I and Beclin1 and decreased p62 levels. α-Mangostin reduced EBI3 and p-STAT3 levels.	SGC7901 and SGC7901/CDDP cells	10, 15, 20, 25, and 30 μM	Incubation (24 and 48 h)	Dose-dependent	[32]
α-Mangostin reduced increases in MMP2 expression from exosomes.	SKOV-3 and TOV-21G cells	12.5 (SKOV-3) and 29.98 (TOV-21G) µM	Incubation (24 h)	Dose-dependent	[67]
α-Mangostin induced oxidative stress, decreased glutathione levels, increased levels of GSSG, and elevated carbonyl protein levels. α-Mangostin induced G2/M phase arrest.	Daoy cells	10, 15, 20, and 40 μM	Incubation (24 h)	Dose-dependent	[61]
α-Mangostin decreased the expression of MMP-2 and MMP-9.	HN-22, HN-30, and HN-31 cells	1–12.20 μM	Incubation (48 h)	Dose-dependent	[50]
α-Mangostin attenuated PSC activation. α-Mangostin inhibited HIF-1α accumulation, suppressed GLI1 expression under hypoxia, and inhibited pancreatic cancer invasion.	PSCs cells	16 μM	Incubation (24 h)	-	[10]
α-Mangostin reduced cell growth and induced morphological changes such as membrane blebbing, cell shrinkage, nuclear condensation, and fragmentation. α-Mangostin reduced the distance between wound edges. α-Mangostin increased the expression of caspase-3, p53, and Bax.	KKU-M214 and Ham-1 cells	1.5, 4, 30, and 60 μg/mL	Incubation (24 and 48 h)	Dose-dependent and time-dependent	[63]
α-Mangostin reduced tumor size, reduced bile duct proliferation, and lowered the expression of PCNA in tumor tissue.	Male Syrian hamsters (90 g, 6 weeks)	100 mg/kg	Oral administration (3 times/week for 21 days)	Dose-dependent and time-dependent	
α-Mangostin reduced Bcl-2 levels and increased cleaved caspase-3 levels. α-Mangostin caused cell cycle arrest at the G1/G0 phase by downregulating cyclin-D1. α-Mangostin inhibited cell migration and invasion. α-Mangostin decreased expression of MMP-2 and MMP-9. α-Mangostin inhibited EMT. α-Mangostin increased E-cadherin levels and decreased expression of vimentin and N-cadherin. α-Mangostin reduced p-Akt (Ser^473^).	BxPc-3 and PANC-1 cells	2, 4, 6, 8, 16, and 32 μM	Incubation (6, 12, 24, and 48 h)	Dose-dependent and time-dependent	[44]
α-Mangostin reduced tumor volume.	Male BALB/c nude mice	50 and 100 mg/kg	Oral administration (5 times/week)	Dose-dependent and time-dependent	
α-Mangostin induced morphological changes indicative of apoptosis, such as cell shrinkage, rounding, membrane blebbing, and nuclear fragmentation. α-Mangostin downregulated the expression of Bcl-2 and upregulated the expression of Bax. α-Mangostin increased expression of p53.	HN-22, HN-30, and HN-31 cells	1–5 μg/mL	Incubation (6, 12, 24, and 48 h)	Dose-dependent and time-dependent	[51]
α-Mangostin reduced sphere formation. α-Mangostin downregulated Notch signaling components, including Notch1, Hes1, and Hey1. α-Mangostin attenuated 5-FU-induced Notch signaling upregulation.	SW620 and HT29 cells	2.5, 5, 10, 20, and 40 μM (SW260) 0.25, 0.5, 1, and 2 μM (HT29)	Incubation (72 h)	Dose-dependent	[36]
α-Mangostin reduced tumor volume. α-Mangostin reduced the proportion of CD133+CD44+ CSCs in excised tumors.	Male BALB/c athymic mice (6 weeks)	5 mg/kg	Intraperitoneal injection (3 times/week)	Dose-dependent	
α-Mangostin reduced RALDH activity in MCTSs.	MCF-7 and MDA-MB-231 cells	5, 10, and 20 μg/mL	Incubation (48 h)	Dose-dependent	[15]
α-Mangostin enhanced p53/DNA damage, Myc/Max, and MAPK/ERK signaling pathways and downregulated the NF-κB pathway.	HCT-116 and CCD-18Co cells	2.5, 5, 7.5, 10, and 12.5 µg/mL	Incubation (48 h)	Dose-dependent	[37]
α-Mangostin decreased expression of caspase-7, caspase-8, and caspase-9, and increased PARP cleavage. α-Mangostin upregulated p53 and Bax expression and downregulated Bid and Bcl-2 expression. α-Mangostin induced the release of cytochrome c from mitochondria into the cytosol. α-Mangostin reduced ERα levels and decreased expression of estrogen-responsive pS2.	MCF-7 and MDA-MB-231 cells	1, 5, and 10 μM	Incubation (48 h)	Dose-dependent	[16]
α-Mangostin reduced GBC cell growth and suppressed colony formation and proliferation. α-Mangostin induced apoptosis and cell cycle arrest.	GBC-SD, HIBEC, and NOZ cells	1, 2, 4, 6, 8, 12, and 16 μM	Incubation (24, 48, and 72 h)	Dose-dependent and time-dependent	[41]
α-Mangostin reduced tumor growth.	Male BALB/c nude mice (4 weeks)	2 mg/kg	Intraperitoneal injection (4 weeks)	Dose-dependent and time-dependent	
α-Mangostin resulted in cell cycle arrest at the G2/M phase. α-Mangostin induced DNA fragmentation and apoptosis.	HCT-116 cells	10 and 20 μM	Incubation (24 h)	Dose-dependent	[62]
α-Mangostin suppressed cell proliferation and decreased colony formation. α-Mangostin elevated expression of Bax, caspase-3, caspase-8, and caspase-9 and decreased expression of Bcl-2. α-Mangostin decreased MMP and increased ROS production. α-Mangostin reduced cell migration and invasion. α-Mangostin decreased expression of phosphorylated PI3K, Akt, and mTOR.	OVACAR-3 cells	5, 25, 50, 100, and 200 μM	Incubation (12, 24, and 48 h)	Dose-dependent and time-dependent	[57]
α-Mangostin inhibited MARK4 kinase activity and decreased cell proliferation. α-Mangostin induced apoptosis and G0/G1 cell cycle arrest. α-Mangostin reduced tau phosphorylation. α-Mangostin reduced ROS levels. α-Mangostin inhibited cancer cell migration.	MCF-7 and HepG2 cells	1–50 μM	Incubation (24 and 48 h)	Dose-dependent	[66]
α-Mangostin induced apoptosis characterized by nuclear condensation and increased sub-G1 fraction. α-Mangostin disrupted mitochondrial function. α-Mangostin altered expression of Bcl-2 and Bak. α-Mangostin collapsed mitochondrial membrane potential. α-Mangostin released cytochrome c and activated a caspase cascade. α-Mangostin inhibited cell migration and invasion. α-Mangostin suppressed MAPK signaling activation.	MG-63 cells	2.5, 5, 10, 15, 20, 30, 40, and 50 μM	Incubation (24, 48, and 72 h)	Dose-dependent and time-dependent	[26]
α-Mangostin inhibited the self-renewal capacity of CSCs. α-Mangostin impaired spheroid and colony formation. α-Mangostin downregulated CSC markers and pluripotency-maintaining factors. α-Mangostin suppressed the Shh signaling pathway. α-Mangostin reduced Nanog binding to gene promoters. α-Mangostin inhibited cell motility, migration, and invasion. α-Mangostin suppressed the expression of EMT markers. α-Mangostin inhibited the transcription and expression of GLI.	AsPC-1 and PANC-1 cells	1–10 µM	Incubation (24 and 48 h)	Dose-dependent	[45]
α-Mangostin inhibited re-adhesion and migration, decreased matrix metalloproteinases MMP-2 and MMP-9 secretion, and reversed EMT phenotypes. α-Mangostin downregulated Akt and ERK pathways.	HepG2 cells	1, 2, 5, 5.5, 7, 10, 14, 15, and 20 μM	Incubation (24 h)	Dose-dependent	[47]
α-Mangostin elevated caspase-3, caspase-8, and caspase-9 activity and cytochrome c level in the cytosol. α-Mangostin induced cell cycle arrest at G1-phase with a reduction in the S-phase population. α-Mangostin inhibited tumor growth. α-Mangostin decreased metastatic expansion. α-Mangostin increased the number of apoptotic cells.	BJMC3879 luc2 and MDA-MB231 cells	4, 8, 12, 16, and 20 μM	Incubation (24 and 48 h)	Dose-dependent	[17]
α-Mangostin reduced the number of dilated lymphatic vessels containing intraluminal tumor cells. α-Mangostin decreased total Akt and p-Akt (Thr^308^) levels.	Female BALB/c mice (6 weeks)	10 and 20 mg/kg/day	Subcutaneously-implanted mini-osmotic pumps (6 weeks)	Dose-dependent	
α-Mangostin inhibited clonogenic potential and induced cell cycle arrest in G1 phase and apoptosis. α-Mangostin activated caspase-3. α-Mangostin inhibited expression of cyclin/CDK proteins (particularly cyclinD1/CDK4). α-Mangostin increased p27 Kip1 expression and decreased cyclins D1 and D3. α-Mangostin decreased cyclin E and p-Rb expression.	LNCaP, PC3, DU145, and 22Rv1 cells	5, 7.5, 10, 15, 20, 25, 30, 35, 40, 60, and 80 μM	Incubation (24 and 48 h)	Dose-dependent	[42]
α-Mangostin inhibited tumor growth.	Male athymic (nu/nu) nude mice (7–8 weeks)	100 mg/kg	Oral gavage (5 times/week)	Dose-dependent	
α-Mangostin reduced MMP-2, MMP-9, and u-PA activities. α-Mangostin inhibited adhesion, migration, and invasion. α-Mangostin inhibited phosphorylation of JNK1/2. α-Mangostin reduced the nuclear levels of NF-κB, c-Fos, and c-Jun. α-Mangostin inhibited NF-κB and AP-1 DNA binding activity.	PC-3 cells	1, 3, and 5 μg/mL	Incubation (12, 24, 36, and 48 h)	Dose-dependent	[73]
α-Mangostin induced autophagy activation in intestinal epithelial cells and apoptotic effect with thapsigargin-like Ca^2+^-ATPase inhibitory effect.	CT26 and Her-2/neu cells	100 and 250 ng/mL	Incubation (4 h)	-	[38]
α-Mangostin reduced ER stress induced by thapsigargin and inhibited eIF2α phosphorylation.	BALB/c mice and C57BL/6 mice (6 weeks)	20 mg/kg	Oral administration (3 days)		
α-Mangostin inhibited PMA-induced cell adhesion, invasion, and migration. α-Mangostin reduced MMP-2 and MMP-9 activities and mRNA expressions. α-Mangostin inhibited FAK and ERK phosphorylation induced by PMA. α-Mangostin inhibited IkBα degradation and p50/p65 nuclear translocation induced by PMA.	A549 and WI-38 cells	1, 2.5, 5, 7.5, 10, 12.5, 15, and 17.5 μM	Incubation (24 and 48 h)	Dose-dependent and time-dependent	[68]
α-Mangostin induced apoptosis.	D-17 cells	5, 10, 15, 20, and 30 μg/mL	Incubation (3, 6, 9, 12, and 24 h)	Dose-dependent and time-dependent	[27]
α-Mangostin reduced clonogenic capacities. α-Mangostin induced G2-M phase cell cycle arrest and apoptosis. α-Mangostin inhibited STAT3 activation. α-Mangostin suppressed the activation of upstream kinases involved in STAT3 phosphorylation. α-Mangostin increased expression of SHP1.	HepG2, Huh-7, SK-Hep-1, and SMMC-7721 cells	1, 2, 5, 10, 20, and 40 μM	Incubation (24, 48, and 72 h)	Dose-dependent and time-dependent	[48]
α-Mangostin inhibited the growth of HCC tumors. α-Mangostin reduced Ki-67 expression, decreased Bcl-2 expression, inhibited STAT3 phosphorylation, and induced SHP1 expression.	Male BALB/c nude mice (6 weeks)	50 mg/kg	Intraperitoneal injection (once/day for 20 days)	Dose-dependent and time-dependent	
α-Mangostin induced cell cycle arrest and decreased the expression of HPV16 oncogenes (E6 and E7) and KCNH1.	C33a, HeLa, SiHa, and CaSki cells	1–10 µM	Incubation (48 h)	Dose-dependent	[29]
α-Mangostin reduced tumor growth and inhibited the expression of E6, E7, and KCNH1 and increased the expression of Ki-67.	Female athymic BALB/c nude mice (6 weeks)	8 mg/kg	Oral administration (4 weeks)	Dose-dependent	
α-Mangostin caused significant changes in nuclear morphology and induced DNA strand breakages. α-Mangostin increased the formation of intracellular oxidative damages. α-Mangostin induced apoptosis in the cells through activation of both extrinsic and intrinsic pathways.	SNUC5 and SNUC5/5-FUR cells	5, 10, 15, and 20 μM	Incubation (12, 24, and 48 h)	Dose-dependent and time-dependent	[59]
α-Mangostin induced autophagy and activated the LKB1/AMPK signaling pathway.	GBM8401 and DBTRG-05MG cells	2.5, 5, 7.5, and 10 μM	Incubation (6, 12, 24, 36, and 48 h)	Dose-dependent and time-dependent	[64]
α-Mangostin inhibited tumor growth. α-Mangostin increased phosphorylation of AMPK and RAPTOR.	Male BALB/cA-ν (*ν*/*ν*) nude mice (6 weeks)	2 mg/kg	Intraperitoneal injection (daily)	Dose-dependent and time-dependent	
α-Mangostin increased miR-143 levels and reduced ERK5 expression.	DLD-1 cells	2, 5, 10, 15, and 20 μM	Incubation (6, 12, 18, 24, 36, and 48 h)	Dose-dependent and time-dependent	[40]
α-Mangostin increased H3K4me2 accumulation and CD86 expression. α-Mangostin inhibited cell migration and modulated the expression of E-cadherin and N-cadherin.	MDA-MB-231 cells	0.62, 1.25, and 2.50 μM	Incubation (24, 36, and 48 h)	Dose-dependent and time-dependent	[18]
α-Mangostin increased caspase-8 and caspase-9 activity and induced apoptosis. α-Mangostin increased cytochrome c release from mitochondria. α-Mangostin induced cell cycle-related gene modulation. α-Mangostin downregulated Akt and NF-κB mRNA expression. α-Mangostin decreased BRAF V600E mutant gene expression. α-Mangostin inhibited Akt expression and p-Akt (Ser^473^) level.	SK-MEL-28 cells	5 and 7.5 μg/mL	Incubation (48 h)	Dose-dependent	[54]
α-Mangostin induced a loss of mitochondrial membrane potential in OSCC cells and triggered the release of cytochrome c from mitochondria. α-Mangostin increased expression of Bak and cleaved forms of caspase-3 and PARP. α-Mangostin induced G1 phase arrest and downregulated CDK/cyclin complex proteins and upregulated the CDK inhibitor p21.	HSC-2, HSC-3, and HSC-4 cells	1–10 μM	Incubation (6, 12, 24, 48, and 72 h)	Dose-dependent and time-dependent	[52]
α-Mangostin caused significant necrosis. α-Mangostin increased caspase activity, particularly caspase-3. α-Mangostin induced cell cycle arrest at the G2/M phase. α-Mangostin upregulated Bcl-2.	SKOV-3 cells	0.305, 0.609, 1.218, 1.827, 2.436, 3.654, 4.873, 6.091, 7.309, and 9.745 μM	Incubation (24, 48, and 72 h)	Dose-dependent	[58]
α-Mangostin reduced the surface rigidity of cells. α-Mangostin inhibited the migration of A549 lung cancer cells.	A549 cells	1–100 µM	Incubation (24, 48, 72, and 96 h)	Dose-dependent and time-dependent	[23]
α-Mangostin inhibited tumor formation and growth, decreased tumor incidence rate and multiplicity, and inhibited DMBA/TPA-induced hyperplasia. α-Mangostin promoted apoptosis and enhanced autophagy.	Female ICR mice (25–30 g, 6 weeks)	5 and 20 mg/kg	Intraperitoneal injection (once/day)	Dose-dependent	[56]
α-Mangostin reduced melanin production. α-Mangostin suppressed tyrosinase activity and downregulated the expression of key melanogenesis-related gene products, including tyrosinase and MITF. α-Mangostin upregulated the phosphorylation of GSK3β and ERK.	B16F10 cells	3, 6, and 9 μM	Incubation (72 h)	Dose-dependent	[55]
α-Mangostin reduced intracellular ROS levels and induced apoptosis. α-Mangostin inhibited the activation of NF-κB and STAT3 signaling pathways. α-Mangostin inhibited IL-6-induced phosphorylation of STAT3 and NF-κB. α-Mangostin suppressed the proliferation of PC cells induced by IL-6. α-Mangostin reduced the activity and expression of MMP9. α-Mangostin increased TIMP-1 levels. α-Mangostin inhibited cyclin D1 and gp130 expression, downstream targets of STAT3, and decreased Bcl-3 levels. α-Mangostin induced cell cycle arrest in the G0/G1 phase and inhibited the invasion and colony formation.	PANC-1, BxPC3, and PL-45 cells	2, 4, 5, 6, 7.5, 10, 12, 15, 16, 20, 24, and 30 μM	Incubation (12 and 24 h)	Dose-dependent	[14]
α-Mangostin inhibited the growth of PC cell-derived orthotopic and ectopic xenograft tumors, reduced tumor volume and weight, and decreased proliferation markers.	Athymic nude mice (6 weeks)	6 mg/kg	Intraperitoneal injection (5 days)	Dose-dependent	
α-Mangostin reduced cell viability, sphere-forming ability, and stemness marker expression. α-Mangostin induced apoptosis and mitochondrial depolarization.	HeLa and SiHa cells	10, 20, and 30 μM	Incubation (24 h)	Dose-dependent	[30]
α-Mangostin reduced tumor growth.	Male BALB/c-nude mice (5 weeks)	40 mg/kg	Oral administration (every 3 days)	Dose-dependent	
α-Mangostin inhibited TPA-induced adhesion, invasion, and migration of MCF-7 cells. α-Mangostin suppressed MMP-2 and MMP-9 expression and activity. α-Mangostin inhibited TPA-induced activation of NF-κB and AP-1, as well as their DNA binding activities. α-Mangostin reduced nuclear levels of NF-κB, c-Fos, and c-Jun, and enhanced IκBα levels.	MCF-7 cells	2, 4, 6, 8, 10, 12, 14, and 16 μM	Incubation (24 and 48 h)	Dose-dependent and time-dependent	[69]
α-Mangostin induced typical apoptotic DNA fragmentation and caspase-3 cleavage. α-Mangostin depolarized mitochondria and led to cytochrome c release. α-Mangostin inhibited Ca^2+^-ATPase activity. α-Mangostin phosphorylated and activated JNK.	PC12 cells	1–100 μM	Incubation (3, 6, 12, and 24 h)	Dose-dependent and time-dependent	[65]
α-Mangostin inhibited migration and invasion. α-Mangostin reversed the LPS-induced upregulation of MMP-9 and MMP-2 expression and downregulation of E-cadherin expression. α-Mangostin suppressed ERK1/2 phosphorylation.	BxPC-3 and MIA PaCa-2 cells	5, 7.5, 10, and 15 μM	Incubation (6, 12, 18, 24, and 48 h)	Dose-dependent and time-dependent	[70]
α-Mangostin reduced volume and increased compactness of spheroids. α-Mangostin inhibited cell motility.	MDA-MB-231 and MCF-7 cells	0.1, 0.5, 1, 5, 10, 15, 20, and 30 μg/mL	Incubation (4, 24, and 48 h)	Dose-dependent	[19]
α-Mangostin inhibited hypoxia-induced ROS formation and VEGF-induced permeability. α-Mangostin attenuated VEGF-induced proliferation and migration. α-Mangostin inhibited VEGF-induced cellular alignment and vascular sprouting in both in vitro and ex vivo assays. α-Mangostin suppressed VEGFR2 and ERK1/2-MAPK signaling.	REC cells	1, 4, and 8 μM	Incubation (24 h)	Dose-dependent	[72]
α-Mangostin inhibited A549 cell migration and increased ROS generation. α-Mangostin modulated antioxidant enzyme activity.	A549 cells	2.5, 5, 10, 25, and 50 μM	Incubation (24 h)	Dose-dependent	[24]
α-Mangostin increased PARP cleavage and induced apoptosis. α-Mangostin decreased Bcl-2 and increased Bax. α-Mangostin inhibited intracellular FAS expression and activity. α-Mangostin affected the phosphorylation of ERK1/2 and Akt. α-Mangostin downregulated FAK phosphorylation.	MCF-7 and MDA-MB-231 cells	1, 2, 3, 4, 6, 8, 10 μM	Incubation (24 and 48 h)	Dose-dependent and time-dependent	[20]
α-Mangostin induced autophagy. α-Mangostin increased expression of LC3II/LC3I and p62. α-Mangostin induced ER stress.	MDA-MB-231 and MCF-7 cells	1, 2, and 4 μM	Incubation (24 h)	Dose-dependent	[21]
α-Mangostin inhibited cell motility, migration, invasion, and adhesion. α-Mangostin downregulated the expression of metastasis-related genes.	SK-MEL-28 and A-431 cells	0.5–2.5 μg/mL (SK-MEL-28) and 0.5–1.25 μg/mL (A-431)	Incubation (24 and 48 h)	Dose-dependent	[71]
α-Mangostin reduced intracellular NAD levels and suppressed NAMPT expression. α-Mangostin increased expression of cleaved caspases. α-Mangostin reduced mitochondrial membrane potential.	A549 cells	2, 4, 6, 8, 10, and 12 μg/mL	Incubation (24 h)	Dose-dependent	[22]
